# Not knowing enough, not having enough, not feeling wanted: Challenges of community health workers providing maternal and newborn services in Africa and Asia

**DOI:** 10.1371/journal.pone.0274110

**Published:** 2022-09-09

**Authors:** Abimbola Olaniran, Aduragbemi Banke-Thomas, Sarah Bar-Zeev, Barbara Madaj

**Affiliations:** 1 Centre for Maternal and Newborn Health, Liverpool School of Tropical Medicine, Liverpool, United Kingdom; 2 School of Human Sciences, University of Greenwich, London, United Kingdom; Jhpiego, UNITED STATES

## Abstract

**Background:**

Community health workers (CHWs) have been identified as a critical bridge to reaching many communities with essential health services based on their social and geographical proximity to community residents. However, various challenges limit their performance, especially in low-and middle-income countries. With the view to guiding global and local stakeholders on how best to support CHWs, this study explored common challenges of different CHW cadres in various contexts.

**Methods:**

We conducted 36 focus group discussions and 131 key informant interviews in Bangladesh, India, Kenya, Malawi, and Nigeria. The study covered 10 CHW cadres grouped into Level 1 and Level 2 health paraprofessionals based on education and training duration, with the latter having a longer engagement. Data were analysed using thematic analysis.

**Results:**

We identified three critical challenges of CHWs. First, inadequate knowledge affected service delivery and raised questions about the quality of CHW services. CHWs’ insufficient knowledge was partly explained by inadequate training opportunities and the inability to apply new knowledge due to equipment unavailability. Second, their capacity for service coverage was limited by a low level of infrastructural support, including lack of accommodation for Level 2 paraprofessional CHWs, inadequate supplies, and lack of transportation facilities to convey women in labour. Third, the social dimension relating to the acceptance of CHWs’ services was not guaranteed due to local socio-cultural beliefs, CHW demographic characteristics such as sex, and time conflict between CHWs’ health activities and community members’ daily routines.

**Conclusion:**

To optimise the performance of CHWs in LMICs, pertinent stakeholders, including from the public and third sectors, require a holistic approach that addresses health system challenges relating to training and structural support while meaningfully engaging the community to implement social interventions that enhance acceptance of CHWs and their services.

## Introduction

Although the global call for health for all through universal health coverage (UHC) continues to gain momentum, equitable access to essential health services remains a challenge in many communities in low-and middle-income countries (LMICs) [1, 2]. Community health workers (CHWs) have been identified as a critical bridge for reaching these communities with essential health services because of their geographical and social proximity to community residents and the cost-effectiveness of engaging them in service delivery [2, 3]. Accordingly, global health stakeholders continue to reaffirm the importance of CHWs in achieving UHC, and CHWs are increasingly taking on critical roles in health service delivery [2, 4, 5]. In LMICs with a triple challenge of a low density of doctors and nurse-midwives, poor national health expenditure, and a high proportion of poor health outcomes, CHWs are considered the ‘golden geese’ needed to make UHC a reality [6–9].

Particularly in maternal and newborn health (MNH), a priority global health challenge for decades, necessitating an increase in the breadth and depth of CHW roles [10, 11]. Given the financial constraints in many LMICs, increased reliance on CHWs often lacks commensurate support with a resultant underperformance, poor retention and professional inertia in which CHWs retain the position without being active [2, 12, 13]. Furthermore, there are concerns that many CHW-delivered interventions in LMICs underperform when scaled to national level from small-scale programmes supported by non-governmental organisations [14, 15]. Therefore, it is vital to fully understand CHW challenges to avoid the mistakes of the 1970s and 1980s, in which many national CHW programmes were abandoned due to underperformance following scale-up [16].

There are a few studies that describe the potential and effectiveness of CHWs to function in MNH [17]. However, to the best of our knowledge, no published study systematically reports the challenges they experience from their point of view and those they intersect with within the health system. This study explored common challenges of different CHW cadres providing MNH services as part of national CHW programmes in LMICs, using their typical roles in MNH as a basis for comparison. It is anticipated that our findings and their implications will guide global and local stakeholders on how best to support CHWs.

## Method

### Study design and setting

This study was part of broader research that used a multiple case study design to explore the functioning of CHWs providing MNH services in LMIC settings. Specifically, this part of the study focused on the challenges of CHWs providing MNH services in sub-Saharan Africa and South Asia from the perspective of the CHWs themselves and the stakeholders who work with them. Other components of the larger study focusing on CHWs’ scope of practice in MNH and factors influencing their motivation and job satisfaction have been published elsewhere [11, 18].

### Selection of study sites

Within the two sub-continents, five countries with public health challenges relating to health workforce shortage, high maternal mortality ratios and high neonatal mortality rates were selected using multistage sampling [19, 20]. The first-stage sampling unit comprised all countries within these subcontinents. Countries with health workforce shortages, high maternal mortality ratios and high neonatal mortality rates within the subcontinents were identified from a limited review of WHO publications. These countries formed the second-stage sampling unit [19, 20]. Subsequently, 11 countries where the Centre for Maternal and Newborn Health at the Liverpool School of Tropical Medicine implemented the ‘Making it Happen’ programme to improve MNH outcomes were drawn out of 47 countries within the second-stage sampling. These constituted the third-stage sampling unit. Using a limited literature review, countries with large-scale CHW MNH programmes were identified from the third-stage sampling unit. In the end, Bangladesh and India (South Asia) and Kenya, Malawi, and Nigeria (sub-Saharan Africa) were identified. The location of ‘Making it Happen’ programme office within each country influenced the selection of specific states/counties/districts as study sites.

### Sampling and study population

Participants were purposively sampled for this study. Stakeholders were eligible if they played an active role in the design/implementation of the CHW programme, were health providers in CHW link facilities, or were community members who supervised CHWs. The sample included CHWs, community health committee members, CHW programme staff, and healthcare professionals whose health-related studies in a higher educational institution range between three and six years. These included doctors and nurse-midwives. We identified the main cadres of CHWs providing MNH services in these countries by reviewing policy documents describing the national CHW programme. These included community healthcare providers (CHCPs), community skilled birth attendants (CSBAs), family welfare assistants (FWAs) and health assistants (HAs) in Bangladesh; accredited social health activists (ASHAs) and auxiliary nurse midwives (ANMs) in India; community health volunteers (CHVs) in Kenya; health surveillance assistants (HSAs) in Malawi; and community health extension workers (CHEWs) and junior community health extension workers (JCHEWs) in Nigeria. Table 1 shows the study countries, locations, CHW cadres and data collected in each country.

**Table 1:** Summary of data collection in study countries.

Country and district/state/county	Primary data collection method and sources (participants)	No of M:F participants	Average no. of years working with CHWs
**Bangladesh:** Dhaka and Sirajganj	• Seven KIIs with seven programme staff	5:2	19
	• Four KIIs with four health professionals	0:4	5
	• three FGDs with 16 community health committee members	16:0	6
	• Eight KIIs with eight CHWs	8:0	4
**India:** Delhi, Maharashtra	• 12 KIIs with 12 programme staff	2:10	8
	• six KIIs with six health professionals	2:4	16
	• Two KIIs and two FGDs with 20 community health committee members	3:19	8
	• 16 KIIs and four FGDs with 51 CHWs	0:67	7
**Kenya**: Nairobi and Kiambu	• Seven KIIs with seven programme staff	3:4	9
	• 13 KIIs with 13 health professionals	3:12	5
	• One KII and four FGDs with 26 community health committee members	18:9	5
	• One KII and 7 FGDs with 54 CHWs	12:43	5
**Malawi:** Lilongwe	• Five KIIs with five programme staff	2:3	11
	• Seven KIIs with seven health professionals	4:3	3
	• Five FGDs with 51 community health committee members	28:23	5
	• Three KIIs and 4 FGDs with 34 CHWs	26:11	11
**Nigeria**: Abuja	• Nine KIIs with nine programme staff	5:4	9
	• Five KIIs with five health professionals	2:3	8
	• One KII and two FGDs with 11 community health committees	8:3	8
	• Nine KIIs and one FGD with 15 CHWs	7:17	9

#### Data collection

We consulted institutions within the study countries and online sources for national policy documents that describe characteristics relating to CHW education, duration of training, selection, and scope of practice. To understand CHW challenges, we conducted focus group discussions (FGDs) and key informant interviews (KIIs) with purposively sampled community and formal health system level stakeholders in each study country between August 2015 and March 2016. Invitation of study participants was done through email, phone calls, and, where possible, face-to-face discussion.

A topic guide was developed to explore CHWs’ activities, including challenges that affect their performance. Relevant revisions were made after pretesting the guide and obtaining inputs from in-country partners. Data collection continued until data saturation was realised [21]. Data saturation was established through an iterative process of preliminary data analysis during data collection. It was noted that data from additional discussions and interviews continued to confirm emerging themes.

### Data management and analysis

To objectively compare ten cadres of CHWs with diverse training duration, they were categorised into Level 1 and Level 2 paraprofessional CHWs based on findings of a systematic review that categorised CHWs based on their education and pre-service training duration. This review described Level 1 paraprofessionals as "individuals with some form of secondary education and subsequent informal training lasting a few days to a few weeks." Level 2 paraprofessionals were described as "individuals with some form of secondary education and subsequent formal training lasting a few months to more than a year" [22]. Subsequently, we explored national policy documents to gain an insight into the selection, scope of practice and primary workstation of each of the ten CHW cadres. We sought to identify patterns between these characteristics and the CHW categories identified in the systematic review. It was anticipated that challenges faced by CHWs might differ based on the CHW category.

A coding template informed by the CHW category was developed in the NVivo 12 software (QSR International, QSR International, Melbourne, Australia), which was used to sort FGD and KII transcript data. Transcripts were read several times to familiarize and understand the various challenges of CHWs. Using thematic analysis [23, 24], we explored the data to understand if and how challenges differed across CHW categories and continents to identify common themes and those unique to the CHW category or continent. Constructs within the data that explain the CHW challenges were used to generate the initial set of codes, which were applied to the transcripts on subsequent readings. The recurring patterns across congruent codes informed the subthemes and themes [24]. Overall, triangulation of information from multiple stakeholder groups allowed the validation of findings.

### Ethics

Liverpool School of Tropical Medicine, Liverpool, United Kingdom, granted full ethical approval (LSTM 15.007). Additionally, ethical approvals were obtained from each of the study countries: Ethical Review Committee, Centre for Injury Prevention and Research, Bangladesh; Institutional Research Ethics Committee (IREC) of The Foundation for Research in Community Health (IREC/2015/11/4/2) Kenyatta National Hospital and University of Nairobi, Ethics and Research Committee, Nairobi, Kenya (P485/07/2015); The College of Medicine Research and Ethics Committee, College of Medicine, Blantyre, Malawi (P.07/15/1765); Federal Capital Territory Health Research Ethics Committee, Abuja (FHREC/2015/01/52/24-08-15). Written consent was obtained from all study participants in English or the local language.

## Results

### Profile of study participants

Table 1 describes the stakeholders who participated in the study across the five countries. In the Asian countries, CHWs providing MNH services were mostly or exclusively female, whereas they were more mixed (male and female) in the African countries. Apart from Malawi, the health professionals were predominantly females, while the community health committee members were mostly male except in India. CHWs included in the study had spent at least four years providing services within the community. The CHWs in Malawi had the most extended work experience of 11 years. Among the other groups of stakeholders, the programme staff had worked with CHWs for the most extended duration except in India, where health professionals had worked with CHWs for 16 years.

### Characteristics of CHWs

Table 2 draws on data from CHW-related national policy documents from study countries [25–33] and transcripts to describe the CHW cadres in the five study countries. All the CHW cadres included in the study had secondary education, but their roles differed by demographic characteristics and country. For example, only female candidates were selected to perform some CHW MNH roles (such as antenatal care) in Asian countries.

**Table 2:** Characteristics of the CHWs in study countries (adapted from Olaniran et al. 2021) [1,8].

Country	*Cadre	**Selection criteria	Pre-service training	Role	Primary workstation	Remuneration type	Career path	Reference
Bangladesh	CHCP	• Motivated community member	Three months	• Support ANC, PNC & neonatal care • Treat mild anaemia • Vaccination • Dispense condoms and oral contraceptives	Facility	Salary + incentive	CSBA	[26]; (Quote 1,2,3,5,6)
	CSBA	• Motivated community member • Prior CHW training • A woman less than 45 years old; • Last child > 1yr • Interested in conducting normal deliveries	Six months	• Support ANC, PNC & neonatal care • Diagnose complications in pregnancy • Vaccination • Normal delivery • Basic newborn resuscitation	Facility	Salary + incentive	Not documented	[26]; (Quote 1,2,3,4,5,6)
	FWA	• Motivated female community member	21 days	• Community mobilization • Support ANC, PNC & neonatal care • Vaccination • Dispense condoms & oral contraceptives • Administer contraceptive injection	Community	Salary + incentive	CSBA	[26]; (Quote 1,2,3,5,6)
	HA	• Motivated community member	21 days	• Community mobilization • Support ANC, PNC & neonatal care • Vaccination • Dispense condoms	Community	Salary + incentive	CSBA	[26]; (Quote 1,2,3,5,6)
India	ASHA	• Female community member • Aged between 25–45 years • Preferably married/widowed/divorced or separated	Eight days	• Community mobilization • Support ANC, PNC & neonatal care • Dispense condoms	Community	Incentive	Not documented	[27, 28]
	ANM	• Female community member	Two years	• Support ANC, PNC & neonatal care • Diagnose complications in pregnancy • Treat mild anaemia • Administer parenteral medications • Vaccination • Normal delivery • Basic newborn resuscitation • Dispense oral contraceptives & condoms • Administer contraceptive injections	Facility	Salary + incentive	Lady health visitor	[29]; (Quote 7)
Kenya	CHV	• Community members • Respected and acceptable Financially self-supported Has leadership qualities	Ten days	• Community mobilization • Support ANC, PNC & neonatal care • Dispense condoms	Community	Allowance + income-generating activities	Not documented	[30, 31]
Malawi	HSA	• Preferably a member of the community	Three months	• Community mobilization • Support ANC, PNC & neonatal care • Vaccination • Dispense condoms	Facility	Salary	Senior HSA	[32]; (Quote 8,9)
Nigeria	JCHEW	• Preferably a member of the community	Two years	• Support ANC, PNC & neonatal care • Diagnose complications in pregnancy • Treat mild anaemia • Vaccination • Dispense condoms	Facility	Salary	CHEW	[33, 34]
	CHEW	• Preferably a member of the community	Three years	• Support ANC, PNC & neonatal care • Diagnose complications in pregnancy • Treat mild anaemia • Administer parenteral medications • Vaccination • Normal delivery • Basic newborn resuscitation • Dispense oral contraceptives & condoms • Administer contraceptive injections • Insert intrauterine device	Facility	Salary	Community Health Officer	[33, 34]

* CHW cadres

**Bangladesh**: Community Health Care Providers (CHCP), Community Skilled Birth Attendants (CSBA), Family Welfare Assistants (FWA) and Health Assistants (HA)

**India**: Accredited Social Health Activists (ASHA) and Auxiliary Nurse Midwives (ANM)

**Kenya**: Community Health Volunteers (CHV)

**Malawi**: Health Surveillance Assistants (HSA)

**Nigeria**: Community Health Extension Workers (CHEW) and Junior Community Health Extension Workers (JCHEW)

**All CHWs had some form of secondary education

CHWs’ pre-service training was between eight days and three years, with the training duration being predictive of other characteristics. For example, CHW cadres with a longer training duration tended to be salaried, work primarily in a health facility, and a clear career path. Additionally, CHWs with a more extended training were expected to perform more technical MNH roles, including managing uncomplicated delivery.

Characteristics of CHWs: table of quotes

**Table:** 

Characteristics of CHWs	
Quote 1	“They are people who have the intention to serve, motivation to serve the people.” Programme staff, Bangladesh
Quote 2	“CHWs should be from the same community” CSBA, Bangladesh
Quote 3	“The FWAs are totally female. HAs are usually female. So CSBA is a combination of the two groups, but most of them are female.” Programme staff, Bangladesh
Quote 4	“They have to be less than 45 years of age; the last child is more than a year old and is interested in performing normal deliveries. You cannot be too tough on the criteria, or you will not find any CSBA.” Programme staff, Bangladesh
Quote 5	“My salary is 10,000 takas per month, and there is no other benefit.” CHCP, Bangladesh
Quote 6	“…but in the family planning side, we have good monetary involvement. If you bring a client for ligation, female sterilisation or male sterilisation, there is involvement of money and who will get how much money.” Programme staff, Bangladesh
Quote 7	“After ANM, she will become a lady health visitor, then she will become block nursing officer at the block level and then public health nurse at the district level.” KII 002, Lady health visitor
Quote 8	A senior HSA is selected on merit due to hard work and served for more than four years and also demonstrated leadership capacity” Programme staff Malawi
Quote 9	“Previously, they would recruit and deploy people within their homes, but these days they recruit and deploy anyhow they want.” HAS, Malawi

### Challenges of CHWs

There were three major themes that captured the challenges that CHWs encounter during service delivery that consequently affect their performance. These were inadequate knowledge, insufficient infrastructural support, and poor acceptance of CHW services. Nonetheless, there were differences in the popularity of these challenges reflecting differences in the CHW category and continent.

#### “Not knowing enough”

Various stakeholders, especially nurse-midwives, expressed concerns over an inadequate breadth and depth of the CHW training curriculum, which constrained the acquisition of new knowledge and, consequently, affected their proficiency in assigned roles. Despite the relatively long training period of Level 2 paraprofessional CHWs to enable them to take on complex roles, including clinical procedures [25, 28, 34], there were concerns about their capacity to comprehend and apply the principles underpinning these clinical procedures. For example, nurse-midwives complained that CHWs’ limited theoretical understanding of the principles made teaching clinical procedures to Level 2 paraprofessional CHWs challenging. A nurse-midwife in Nigeria explained, *"And you know that this medical line*, *sometimes it will be very hard if you don’t know the theoretical aspects of it*, *for you to be good in practical*, *it goes together*. *When you [CHW] know the theory*, *when they teach you the practical*, *you will be able to understand*.*"* Similarly, the different stakeholders acknowledged the inadequacy of Level 1 paraprofessional CHWs and explained how this affected their health education role. A midwife in Kenya narrated how limited understanding shapes outcomes of health education sessions: "*Most of them find it very challenging to go there and explain a certain method [contraceptives] because they don’t have most of the information…they are asked some very difficult questions like side effects so that they can assure them [service recipients] maybe it is a normal side effect*. *Maybe some mothers have experienced it*, *and that is why they are changing to another method*."

Reasons advanced for inadequate CHW knowledge fell under four categories:

*a*. *Limited training opportunities for CHWs in remote regions*: CHWs providing services in remote areas complained of limited training opportunities. CHWs in rural and remote areas explained that CHWs in the suburban areas were attending training sessions at their expense because those in the suburban areas were more visible and geographically closer to the district/state programme office, where training invitations were prepared and mailed. This challenge was more popular in the African study countries and explained by a CHW in Malawi: "*Sometimes the people from the facility [suburban areas] would go to a workshop on behalf of the other people without their knowledge pretending to be from these areas [rural areas] making us miss information*. *In the end*, *the HSAs and the community will suffer*."
*b*. *Non-availability of equipment for knowledge application*: According to CHWs, the non-availability of MNH-relevant equipment and tools prevented them from translating their theoretical to experiential knowledge. A CHW in Nigeria talked about lacking requisite equipment and commodities for applying knowledge gained from a training session, thereby leading to progressive depreciation of newly acquired knowledge: *"In that training*, *we were able to learn so many things*, *but coming down to our facility*, *those things [equipment] are not here for us to put them to practice the way we saw them there [at the training]*. *So*, *the knowledge begins to die gradually*. *But in fact*, *that programme [training] was very*, *very interesting*.*"*
*c*. *Long stretch theoretical training without community-level practice between modules***:** There were insinuations that the approach to training Level 1 paraprofessional CHWs in Africa may be contributing to poor knowledge retention. According to the training manual [29], the entire training curriculum was delivered within a few days to weeks rather than spacing training days. This approach deprived CHWs of the opportunity to practise new concepts and gain experiential knowledge before learning new ones. A programme staff in Kenya explained, "*…whatever you are to train them for can be done in a day*, *a day each during the six weeks rather than you do it in three days or five days at a stretch… when you go for one week you are giving these CHVs so many things some of them will not practise them*." In contrast, Level 1 paraprofessional CHWs in South-East Asia (ASHA) undergo training that is spaced-out and assessed a few months after pre-service training for gain in experiential knowledge and to determine if they have been practising the knowledge gained from the previous training. This assessment would qualify them for additional training, as explained by a programme staff in India: *"We assess the knowledge level of ASHAs from the induction training*. *We identify ASHAs who have been practising the things because they recall them*, *and those who do not practise can’t recall them and may not continue for further training*. *For skills*, *we ask them*, *show me how you measure temperature*, *how do you wrap the baby…"*
*d*. *Proliferation of roles limiting proficiency in assigned roles*: Another threat to knowledge and skill retention was the additional roles assigned formally or informally to CHWs by governmental and non-governmental organisations, thereby preventing them from attaining proficiency in previously assigned roles. A programme staff in Bangladesh explained, "*Whatever new thing that is coming to our community*, *this will be given first to the family welfare assistants [CHW]*. *She is given IMCH [Integrated Maternal and Child Health] training; she is given nutrition training*. *They have to know everything because they are the door worker*… *And […] they are only involved with our family planning services*. *One person cannot do the jobs of many people*. *It is a challenge for them*."

#### “Not having enough”

Across study countries, CHWs were challenged by insufficient infrastructural support, which negatively affected service delivery and coverage, but the popularity of this challenge varied with CHW category and primary workstation:

a. *Lack of accommodation for Level 2 paraprofessional CHWs resulted in shorter working hours*: Level 2 paraprofessional CHWs who may not be from the community served were often challenged by a lack of habitable accommodation within the community. As explained by various stakeholders, there were remote communities with low literacy rates with hardly any community member with the required academic qualification for the CHW training school. To ensure that health facilities in these areas were operational, CHWs from other communities were often posted to provide services and were expected to make their accommodation arrangement. This challenge was mainly in the African study countries where being a resident or in marriage to a community member was not a requirement for CHWs, unlike South-East Asian study countries (Table 2). A programme staff in Nigeria explained that CHWs may not be from the community served, "*There are some communities that don’t have [individuals with requisite educational qualification for CHW training school]*. *They need a health facility*, *and the government has made provision for a health facility there*. *So*, *if they don’t have*, *you have no choice but to post somebody [CHW] from elsewhere*". The CHWs reported that they often found it challenging to reside within the community. Therefore, they resorted to shortening their working hours to create adequate travel time to their residence outside the community and limit their availability for emergencies. Another programme staff in Nigeria submitted that "*Most of the facilities are not offering 24-hour services because they don’t have the manpower*, *as the staff don’t have accommodation to stay in the community*, *but delivery does not tell you when it will come; it can come at any time*.*"* Still, CHWs were often discouraged from staying overnight at the health facility because of security challenges such as hoodlums’ attacks. A community health committee member in Nigeria explained, *"This building behind was built to accommodate the health workers to stay*, *to be able to work at night*, *but the abattoir hoodlums did not allow them to*. *All these doors were vandalised*.*"*
b. *Inadequate transportation support with consequent lower service coverage and additional work pressure*: CHWs complained of the bad roads leading to hard-to-reach communities. They narrated how they would walk long distances to deliver services thereby increasing their non-productive time and lowering service coverage. Worse affected were the Level 1 paraprofessional CHWs who are expected to deliver services through home visits. A Level 1 paraprofessional CHW in Bangladesh lamented, *"We face problems carrying logistics*. *We do not have any vehicle*, *and in many communities*, *there is no road to reach them*, *so we need to carry the logistics and medicine on our own"*. Furthermore, poor transportation support caused additional work pressure for CHWs who were expected to accompany women in labour to the hospital but were forced to conduct unsupervised deliveries (despite not having any midwifery training) when the women progressed to an advanced stage of labour while walking to the health facility. In Malawi, a community health committee member narrated, "*The problem that we have here is transport*. *This sometimes makes women deliver in the bush on their way to the hospital*."
c. *Poor availability of supplies limits service coverage*: Across study countries, there were complaints about the non-availability of work tools such as gumboots and umbrellas, which limited their capacity for service coverage during the rainy season, especially among Level 1 paraprofessional CHWs whose role largely depends on conducting home visits. A programme staff in Kenya explained, *"We have no control over the weather*, *but what if they were provided with gumboots*, *raincoats*, *and an umbrella*, *how nice would this have been*."

#### “Not feeling wanted”

Various stakeholder groups underscored the importance of social acceptance in CHW service coverage. Still, they clarified that this was not guaranteed due to socio-cultural beliefs, the influence of demographic factors and visit time conflicts:

a. *Local socio-cultural beliefs influenced health-seeking behaviour and*, *consequently*, *health outcomes*: Across study countries, service recipients prioritised or preferred spiritual to clinical solutions for medical conditions. This sometimes resulted in non-compliance with health education sessions delivered by CHWs. A CHW in India explained, *"Among some nomadic castes*, *like Nandiwale*, *they don’t come forward to take injection [anti-tetanus] unless they perform God’s ritual*. *They come at a later stage*, *such as in the 7th or 8th month of pregnancy…they used to deliver at home*.*"* Similarly, there were occasions when non-compliance and poor health-seeking behaviour resulted in poor health outcomes. A CHW in Kenya narrated, "*There is a woman who delivered at home*, *and the baby died when we reached there*, *and we wanted to take the mother to the hospital [but] they refused and wanted to pray for her*. *So*, *they started praying and eventually the mother died*."
b. *Gender difference between CHWs and their service recipients affected CHW social acceptance*: Across the study countries, participants reported preference for MNH care provided by female CHWs, reflecting the socio-cultural norms guiding male-female interactions. In Kenya, a programme staff explained, *"Even men were not very comfortable with male CHVs [CHWs] coming to talk to their women*, *sometimes in the houses… but prefer if it were women…"* Nonetheless, male CHWs in African study countries provided MNH services to female service recipients, including management of labour and delivery, unlike in South-East Asian study countries. Consequently, there was poor social acceptance and utilisation of the MNH services provided by male CHWs as explained by a programme staff in Nigeria. *“Because in the northern part of Nigeria*, *any labour room that is managed by a male*, *especially in the communities*, *you won’t see anybody [pregnant women]*, *they will continue seeing their TBAs*.*"*
c. *Intra-community differences in language/dialect limited communication*: Across study countries, there was a diversity in languages and dialects within communities, which affected communication. For example, in India, a lady health worker explained the difficulty CHWs experience while interacting with individuals who may not speak a popular national language apart from their local dialect. She stated: "*Although ASHA is from the same community*, *but every 12 km*, *the language differs*, *maybe regarding tone or meanings*. *Some words differ in meanings*, *though they are commonly used*. *So*, *ASHA has to take care of all these things*, *especially when interacting with people from the low educational background or illiterate community*."

Furthermore, the age difference between CHWs and their service recipients also contributed to the communication barrier faced by CHWs, as the vocabulary and choice of words tended to vary across age groups. A community health committee member in Kenya explained, *"We have an age barrier; for example*, *if they [CHWs] go to a young girl’s house*, *sometimes*, *they want information from her*. *There are some words she uses that they don’t understand*, *there is a small barrier*.*"*

d. *Undue familiarity and short training duration resulted in CHW services being undervalued*: In contrast to Level 2 paraprofessional CHWs, Level 1 paraprofessional CHWs with shorter training and predominantly community members were often perceived as lacking the requisite proficiency to perform their assigned roles. This was partly explained by the perceived inadequacy of their training and undue familiarity from being co-community members. A programme staff in India explained that since ASHAs *"belong to that village*, *community members are of the perception that she won’t know anything*.*"*
e. *Time conflict with the daily routine of service recipients*: Across the study countries, CHWs complained that the timing of community clinics and home visits often conflicted with recipients’ routine schedules. Particularly, female Level 1 paraprofessional CHWs complained that their domestic responsibilities prevent them from conducting early-morning home visits. For instance, ASHAs in India explained that they were expected to do their domestic chores before leaving their homes in the morning, which often meant they did not leave home early enough before service recipients leave for their farms or income-generating activities: *"…if you visit the community before 11 o’clock it is only then that you can meet people*. *If you go after that*, *you find all the houses locked because everybody goes to the farm*. *How can we go to visit them before 9 o’clock because we have to look after our household chores also*?*"*.

## Discussion

This study explored and distilled the challenges of various CHW cadres drawing on diverse perspectives of various community health stakeholders. Our findings show how challenges differed by CHW category, but were consistent between the countries and continents. Our findings also highlight how inadequate knowledge, structural support, and social acceptance limit CHW performance. However, we acknowledge that some challenges may reflect poor health systems in LMICs with CHWs disproportionately affected, leading to suboptimal performance.

Our study underscores how inadequate knowledge constrained Level 2 paraprofessional CHWs in performing clinical procedures and Level 1 paraprofessional CHWs in health education. Given the global shift in focus from increased access to improved quality of care [35], our findings raise critical questions about the adequacy of CHW competency to perform assigned roles and underscore the need to overhaul CHW training. Notably, there is a need to revise the training approach of Level 1 paraprofessional CHWs to consolidate knowledge and skills before exposure to new training. Furthermore, these CHWs should be assessed based on the achievement of requisite competencies rather than training duration. Evidence from high-income countries suggests a more efficient model of training could be applied with assessments including observation of skills and review of activity case logs used to inform re-licensing and continued education of CHWs [36, 37]. Consistent with our findings, literature on CHWs in LMICs [38, 39] shows that many governmental and non-governmental organisations continue to add tasks to CHWs’ job descriptions and consequently expand their training curriculum at the expense of knowledge and skill consolidation. Critical policy decisions have to be made on the breadth and depth of CHW training and assigned roles. Stakeholders may borrow ideas and lessons from medical professions in creating specialist CHW roles such as CSBAs in Bangladesh with depth in a subject area and generalist CHW roles with a breadth across subject areas at the expense of depth. However, rigorous research is needed to establish the comparative effectiveness between generalist and specialist CHWs to guide implementation.

Our study highlights the critical role of structural support, including equipment and supplies. First and in line with existing literature [40, 41], equipment and supplies are key prerequisites for translating theoretical to experiential knowledge, thereby enhancing retention and preventing depreciation of newly acquired competency due to non-application. Literature is replete with documentation of CHWs lacking requisite equipment, especially in LMICs [40, 42, 43]. Hence, programme planners need to be intentional about ensuring that CHW capacity-building interventions transcend training to ensure the availability of requisite equipment. Second is the effect of the non-availability of structural support on service coverage. Our study illustrates how CHWs continue to work on the sharp end of the health system with poor working conditions, including inadequate supplies, transportation, and accommodation challenges resulting in poor service coverage. With less than a decade away from the 2030 target year set for achieving the SDGs (including health-related goals), the United Nations has expressed concerns that universal health coverage (UHC) will not have been achieved for up to one-third of the global population as about half of the world’s population still lack access to essential health services [44]. Hence, there is a need to support CHWs in providing basic health services especially to individuals in hard-to-reach communities in LMICs who are unlikely to seek facility-based care because of logistic challenges [45]. Despite robust evidence of CHW effectiveness, public funds are often channelled disproportionally to high-end care at secondary and tertiary levels [46, 47]. Similarly, national investments in CHW programme are only a small fraction of primary health care-directed funds, while only 2.5% of official development assistance for health were specifically channelled to CHW programmes in the last decade [45]. A recent estimation shows that an additional $2 billion is needed annually to build and strengthen community health systems in sub-Saharan Africa in order to meet global health objectives [48]. Stakeholders therefore need to be intentional in funding and implementing CHW training and support, particularly those in hard-to-reach areas where CHWs are the sole health care providers and referrals outside these geographically isolated regions may be too cumbersome and unfeasible [49].

Our study suggests that CHWs are doubly rejected. First is a covert health system rejection by nurse-midwives as the perception of CHWs “not knowing enough” was largely popular among nurse-midwives. In line with the literature [50, 51], the perception of nurse-midwives may be partly explained by professional rivalry, philosophical differences over health practices and tensed relationship between midwives and CHWs which could be addressed by a strengthened health workforce governance. Second is a relatively overt rejection by the community members, which raises the concern that being respected by community members does not guarantee that CHWs and their services would be accepted and valued. In our study countries, acceptance was shaped by socio-cultural beliefs, perception of CHW competency, differences in demographic characteristics between CHWs and service users, and time conflicts with a home visit. Stakeholders are cautioned against viewing all challenges of CHWs through the health system building block lens only as there is a need to understand and address the social drivers of their work. For example, the negative influence of socio-cultural beliefs on acceptance of CHW services was noted in all study countries and has been documented in the existing literature which cuts across country income status. Globally, there is a need to strengthen CHW cultural competencies and guide them in navigating the inherent tensions between their role identities as health workers and cultural consonance in which they are expected to act as "insiders" who are co-custodians of local socio-cultural beliefs. Consequently, they are treated as "outsiders" when they express a preference for "medicalised" approach to disease management [52, 53]. Furthermore, CHW sociodemographic characteristics such as sex, age, and residence shape social acceptance.

Contrary to social norms in which there is a preference for MNH services delivered by female CHWs, male CHWs provide these services in the African study countries. Nonetheless, there are arguments for and against the provision of MNH services by male CHWs. On the one hand, it will promote male partner involvement in MNH care [54]. On the other hand, engaging male CHWs may reinforce gender norms that encourage male dominance in issues relating to women’s reproductive health [55]. Hence, national governments in Africa need to transcend informal gendered division of labour [56] to formally assign MNH roles to CHWs in a way that fosters male partner involvement while preserving the woman’s autonomy in decision-making relating to her health. In addition to gender, our study highlights the varied influence of other sociodemographic factors. For example, age differences tended to limit communication between CHWs and the service recipients as colloquialisms and slang varied between age groups. Research shows that women within the reproductive age group prefer women within their age bracket when discussing sexual and reproductive health issues as demographic similarities aid communication [57, 58]. Communication barrier due to age difference is not unique to LMICs [59]. A global policy review should consider the demographics and unique needs of the target population in developing CHW selection criteria.

In contrast to global literature [60, 61], our findings did not identify supervision as a key challenge of CHWs. Future research may explore CHWs’ experiences to understand why supervision is a challenge in some settings but not others.

### Strengths and limitations

This study makes vital contributions to the existing body of knowledge relating to the challenges of CHWs. To our knowledge, this is the first qualitative study exploring challenges faced by CHWs providing MNH services through the perception of diverse stakeholder groups in five countries across two continents, thereby enabling comparison across contexts.

Nonetheless, certain study limitations should guide the interpretation of our findings. First, this study focused on CHWs providing MNH in five LMICs, and we cannot guarantee that findings reflect a complete list of challenges that will apply to CHWs providing other services or MNH services in high-income settings. Second is the concern about the data’s contemporary relevance as it was collected in 2015/2016. Still, many key findings appear to be consistent with recent literature from diverse settings.

## Conclusions

Addressing CHW challenges require further attention. As key stakeholders continue to position CHWs to improve access to health services among community served, there is an urgent need to address the factors influencing their performance and better position them to meet the community needs. CHW programmes should consider a holistic approach that entails addressing health system factors and social interventions to improve CHW acceptance within the community.
